# Evaluation of prognostic factors in patients undergoing first-line chemotherapy for advanced biliary tract cancer: a retrospective analysis from a South American cancer centre

**DOI:** 10.3332/ecancer.2022.1345

**Published:** 2022-01-17

**Authors:** Tiago Cordeiro Felismino, Felipe Marcio Araujo Oliveira, Camilla Albina Zanco Fogassa, Silvia Nardozza Santerini, Victor Hugo Fonseca de Jesus, Rachel Simões Pimenta Riechelmann, Felipe José Fernandez Coimbra, Celso Abdon Lopes Mello

**Affiliations:** 1 Clinical Oncology Department, AC Camargo Cancer Center, Rua Professor Antonio Prudente 211, São Paulo, 01509-001, Brazil; 2 Surgical Oncology Department, AC Camargo Cancer Center, Rua Professor Antonio Prudente 211, São Paulo, 01509-001, Brazil; a https://orcid.org/0000-0002-1055-8118

**Keywords:** biliary tract neoplasms, prognosis, chemotherapy

## Abstract

**Introduction:**

Biliary tract cancers (BTCs) are rare tumours with regional differences. Prognostic factors are poorly understood. Gemcitabine + platinum (GP) is the standard first-line chemotherapy in metastatic patients. We aimed to search for prognostic factors in patients with advanced disease in a cancer centre in South America.

**Methods:**

We conducted a retrospective analysis of patients with advanced BTC treated with chemotherapy. Variables were age (< or ≥70 years), Eastern Cooperative Oncology Group (ECOG) performance status (0/1 versus 2/3), gender, primary site (intrahepatic (IHC), extrahepatic (EHC), gallbladder (GB)), staging (locally advanced versus metastatic), metastatic sites, albumin (>3.5 g/dL versus <3.5 g/dL), biliary obstruction and first-line chemotherapy (GP, 5FU-based or single-agent). Cox regression method was used to explore factors.

**Results:**

From 2010 to 2017, 104 patients were included. Median age was 62 years (32–86) and 22.1% were older than 70 years. Most patients had ECOG performance status 0/1 (63.4%), were female (51.9%) and were metastatic (82.7%). Bone metastases were found in 19.2%. Primary IHC, EHC and GB were 54.8%, 36.5% and 8.7%, respectively. GP was used by 79.8%. Median follow-up was 32.4 months. Median overall survival (mOS) was 11.4 months. In univariate analysis, male (*p* = 0.007), albumin < 3.5 g/dL (*p* = 0.001), biliary obstruction (*p* = 0.006), 5FU-based (*p* = 0.006) and single-agent (*p* < 0.0001) were associated with worse OS. ECOG performance status 2/3 (*p* = 0.058) and bone metastases (*p* = 0.051) were marginally related. In multivariate analysis, male (*p* = 0.003), bone metastases (*p* = 0.023), biliary obstruction (*p* = 0.001), 5FU-based (*p* = 0.016) and single-agent (*p* = 0.023) were independently associated with inferior OS.

**Conclusion:**

In this retrospective study, we observed that male patients, bone metastases, biliary obstruction and regimens other than GP had worse survival. Larger studies should be conducted to confirm our findings.

## Introduction

Biliary tract cancers (BTCs) are a rare group of malignances [1]. Main subsites are intrahepatic (IHC), extrahepatic cholangiocarcinoma (EHC) and gallbladder (GB) cancer. Incidence is higher in China and southeast Asia [2]. Recently, an increase in the incidence of IHC has been described [3]. However, the incidence of GB cancer has decreased, mainly due to the increase in cholecystectomy. In Latin America, Bolivia has the highest age-adjusted incidence rate for both sex (14.0/100,000) followed by Chile (9.3/100,000) and Peru (4.8/100,000) [4, 5]. The regional differences observed in incidence are mostly due to differences in environmental exposures to various chemicals, genetic predisposition and regional intrinsic risk factors that predispose to carcinogenesis [4]. In the metastatic setting, prognosis is poor. Gemcitabine plus Cisplatin (GC) is considered the standard therapy in the first-line setting based in the ABC-02 trial. Overall survival (OS) reached 11.7 months in the doublet regimen, compared to 8.1 months in gemcitabine monotherapy arm [6]. Recently, ABC-06 showed superiority of FOLFOX over best supportive care in second-line patients [7]. Main prognostic factors are poorly defined due to the rarity and heterogeneity of this disease. Herein we aim to describe main prognostic factors for metastatic BTC in a South American cohort of patients treated at a large cancer centre, who received first-line chemotherapy.

## Methods

We conducted a retrospective analysis of patients treated at a cancer centre in Brazil, from 2010 to 2017. Patients were included if they were 18 years of age or older, had histologic diagnosis of metastatic, recurrent or locally advanced unresectable BTC (IHC or EHC and gallbladder cancer). All patients were deemed eligible to receive first-line chemotherapy by their treating physician. Exclusion criteria were absence of histologic confirmation, absence of data regarding systemic treatment and mixed histologies (hepatocellular-cholangiocarcinoma). Medical files were used as source of information. This study was approved by the local Ethics Committee (2680/19).

### Statistical analysis

Descriptive statistics were used for main demographic characteristics. Survival curves were estimated using Kaplan–Meier method and compared with log-rank test. OS was defined as date of first chemotherapy cycle and death from any cause. Progression-free survival (PFS) was defined as date of first chemotherapy cycle and disease progression or death from any cause. Radiologic assessments of response and clinical benefit were performed according to local guidelines, typically by means of clinical examination every 2 weeks and computed tomography or magnetic resonance imaging every 8 weeks. Progressive disease was identified by the treating physician.

*X^2^* tests were used to analyse categorical variables distributions between genders. To evaluate prognostic factors, univariate and multivariate analysis were performed using the Cox regression method. Age (<70 years versus >70 years), gender, staging (locally advanced versus metastatic), primary site (EHC versus IHC versus GB carcinoma), Eastern Cooperative Oncology Group (ECOG) performance status (0–1 versus 2–3), bone metastasis, baseline Carbohydrate Antigen 19-9 (CA19-9), albumin (<3.5 g/dL versus > 3.5 g/dL), biliary obstruction and first-line treatment (gemcitabine + platinum (GP) versus 5-Fluorouracil (5-FU) based versus monotherapy) were used in the univariate model. All tests were considered statistically significant with a two-sided *p* value of < 0.05. Statistical analysis was performed with IBM SPSS 20.

## Results

Between July/2009 and March/2017, 104 patients were identified. Main demographics are shown in Table 1. Median age was 62 years (32–86). Patients older than 70 years were 23 (22.1%). Male/female proportion was 50 (48.1%) and 54 (51.9%), respectively. Primary site was IHC in 57 (54.8%), EHC in 38 (36.5%) and GB in 9 (8.7%). Metastatic/locally advanced unresectable were 86 (82.7%) and 18 (17.3%). ECOG performance status 0/1 and 2/3 were present in 66 (63.5%) and 37 (35.6%). Main sites of metastasis were liver in 67 (64.4%), distant lymph nodes in 26 (25%) and bone in 20 (19.2%). Median baseline CA19-9 was 185 U/mL (0.6–34.833). Biliary obstruction at diagnosis of advanced disease was present in 26 (25%).

Regarding first-line chemotherapy (Table 2), GP, 5-FU based and monotherapy were used in 83 (79.8%), 12 (11.5%) and 9 (8.7%), respectively. GP combination was 88% Cisplatin and 12% Oxaliplatin. FOLFOX/CAPOX and FOLFIRINOX were used in ten and two patients, respectively. Gemcitabine and Capecitabine monotherapy were used in 6 and 3 patients, respectively. Fifty-nine (56.7%) patients underwent second-line chemotherapy. Main second-line regimens were FOLFIRI (33.9%) and FOLFOX (32.2%).

Numerically, more female patients used GP as first-line (85.2% versus 74%, *p =* 0.32). Access to second-line was not statistically different among gender (59.3% for female versus 54% for male, *p =* 0.69).

### Survival

**Figure 1.**
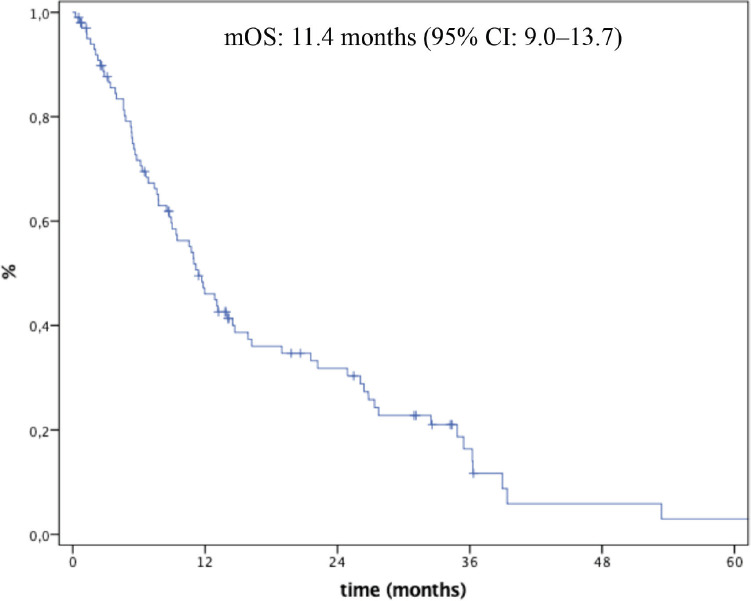
Overall survival.

Median follow-up time was 34.2 months (95% CI: 29.9–38.4). At time of analysis, seventy-six (73.1%) patients had died. Median OS (mOS) was 11.4 months (95% CI: 9.0–13.7) (Figure 1). Median progression-free survival in first-line was 5.6 months (95% CI: 4.0–7.2).

In univariate analysis, male patients (hazard ratio (HR): 1.88 (95% CI: 1.19–2.98); *p* = 0.007), albumin < 3.5 g/dL (HR: 4.73 (95% CI: 1.96–11.4); *p* = 0.001), biliary obstruction (HR: 2.15 (95% CI: 1.24–3.71); *p* = 0.006), 5FU-based (HR: 2.64 (95% CI: 1.31–5.30); *p* = 0.006) and single-agent (HR: 6.27 (95% CI: 2.36–16.6); *p* < 0.0001) were associated with worse OS. Bone metastases (HR: 1.82 (95% CI: 0.99–3.34); *p* = 0.051) and ECOG performance status 2–3 (HR: 1.58 (95% CI: 0.98–2.53); *p* = 0.058) were marginally associated with prognosis.

**Figure 2.**
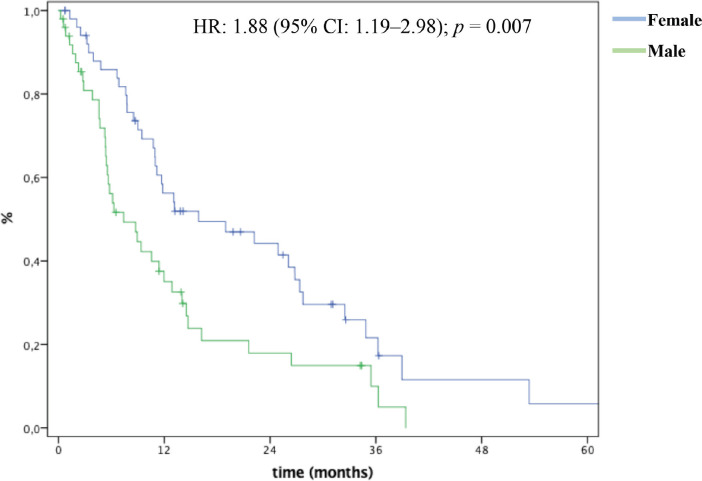
OS by gender.

In multivariate analysis, male patients (HR: 4.18 (95% CI: 1.62–10.8); *p* = 0.003), bone metastases (HR: 3.53 (95% CI: 1.18–10.5); *p* = 0.023), biliary obstruction (HR: 4.28 (95% CI: 1.75–10.440; *p* = 0.001), 5FU-based (HR: 6.24 (95% CI: 1.41–27.6); *p* = 0.016) and single agent chemotherapy (HR: 7.22 (95% CI: 1.30–39.8); *p* = 0.023) were significantly and independently associated with inferior OS (Table 3). mOS was 15.9 months and 7.3 months for female and male patients, respectively (Figure 2).

## Discussion

BTC is a rare gastrointestinal neoplasm but with increasing incidence over the past years, especially for IHC [3]. Surgery is the only curative procedure for a minority of patients who present with localised disease [8]. Survival of patients undergoing palliative chemotherapy for metastatic or irresectable disease is still poor [9]. Any effort to better understand this pathology is worth done. Our study confirmed the survival rates observed in main prospective trials in the metastatic scenario. In our multivariate model for OS, gender, presence of bone metastasis, biliary obstruction and first-line chemotherapy regimen other than GC were correlated to inferior OS.

Our data bring relevant information about two aspects of metastatic BTC. The first is regarding the standard first-line treatment. Patients treated with GP regimens presented a more favourable outcome. GP was superior to Gemcitabine in the Phase III ABC-02 study and was established as the standard of treatment first-line therapy for metastatic biliary tract carcinomas [6]. On the other, based on prospective trials, Gemcitabine plus Oxaliplatin (GemOx) is considered an alternative regimen for first-line [10, 11], although direct comparison of GP and GemOx has not been conducted in a randomised trial.

Recently, the ABC-06 trial established FOLFOX as a new standard for second-line treatment of BTC [7]. Based on the good toxicity profile of FOLFOX and the activity of fluoropyrimidine for gastrointestinal carcinomas in general, this regimen is employed in the first-line for patients with metastatic BTC in specific situations. In 2017, Schinzari *et al* [12] published the results of the phase II trial comparing FOLFOX4 with de Gramont regimen. In this trial, FOLFOX4 had superior PFS (5.2 versus 2.8 months (HR: 0.47 (95% CI: 0.25–0.89); *p* = 0.0031) and OS (13.0 versus 7.5 months (HR: 0.31 (95% CI: 0.15–0.63); *p* = 0.0013). However, in our study, 7.6% of patients were treated with FOLFOX in first-line and despite the small sample, outcomes were worse than those of patients treated with Gemcitabine combinations, reinforcing the role of GP. Clinical reasons not to employ GP were not accurately assessed in our analysis.

Second, female gender was significatively associated with better survival. We found that survival was almost doubled among female patients. In analogous tumours like metastatic pancreatic cancer, gender is a controversial prognostic factor [13, 14] . However, one retrospective analysis suggested that gender may influence responses to FOLFIRINOX [15].

Clinical data endorses our findings. Bridgewater *et al* [16] described main prognostic factors in ABC-02 patients and in an international dataset. Male patients were identified as having worse OS (HR: 1.28 (95% CI: 1.01–1.60); *p =* 0.037). Baton *et al* [17] published a series of 59 hilar cholangiocarcinoma patients who underwent resection. Male gender was related to poor OS (HR: 5.4 (95% CI: 2.2–13.5); *p* = 0.0002). Another retrospective large series from Korea with 740 BTC patients [18] also showed worse survival for male patients (HR: 0.83 (95% CI: 0.69–0.99); *p =* 0.04).

Some aspects of BTC regarding access to therapeutics may help clarify this difference. Undergoing second and further lines of treatment is probably a strong prognostic factor. In a large study of second-line therapy, out of 378 patients that received first-line chemotherapy, only 96 patients (25%) received second-line. Female/male ratio was 31% and 21%, respectively (*p* = 0.03) [19]. In our data, second-line therapy was evenly distributed between genders.

Currently, it is clear that there are striking differences in gastrointestinal cancer incidences among male and female patients. These differences are in part explained by exposition to risk factors such as smoking [20]. Nevertheless, host factors probably play an important role in incidence variations. As an example, androgen levels may be related to the higher incidence of oesophageal adenocarcinoma [21]. However, little is known about gender as a prognostic factor in gastrointestinal malignancies. For instance, in gastro-oesophageal cancer, The Cancer Genome Atlas Program (TCGA) revealed a higher rate of microsatellite instability (MSI-H) among women [22]. It is clear now that MSI-H patients have a better prognosis [23]. In colorectal cancer, women have a higher proportion of right-sided tumours which are linked with poor outcomes [24, 25].

Drug effects are also variable between genders. 5-FU based chemotherapy tends to be more toxic in women. A polled analysis of more than 28,000 patients, demonstrated that neutropenia and gastrointestinal toxicity were more likely to affect female patients [26]. Benefit of immunotherapy also seems to vary according to sex. A large meta-analysis with 11,351 patients with different types of advanced cancer concluded that the magnitude of treatment was greater among men. HR was 0.72 and 0.86 in male and female patients, respectively, in the comparison of immune checkpoint inhibitors and control groups [27].

Finally, it is noteworthy that we have a growing body of evidence regarding molecular biology of BTC. Fibroblast Growth Factor Receptor (FGFR) fusions and Isocitrate Dehydrogenase (IDH) mutations are among targetable alterations in this scenario. Clinical trials have shown benefit of targeted therapies for such patients [28, 29]. Describing main characteristics of these patients, including eventual gender differences, will be paramount to enlighten these findings.

## Conclusion

In summary, our data identified prognostic factors related to outcome in metastatic BTC. Although FOLFOX has become the standard on the second-line, in our series patients receiving first-line treatment other than gemcitabine combination had worse prognosis. We also showed that bone metastasis, biliary obstruction and male gender were correlated with worse prognosis. These data could be useful for future trials in selecting patients with higher risk to more intensive and precise therapy. Further studies and multicentric collaborations are fundamental to validate our findings and also to understand the biology of this rare neoplasm.

## Funding

**None.**

## Conflicts of interest/competing interests

The authors declare that they have no conflicts of interest.

## Ethics approval

Approved by local ethics committee (2680/19).

## Consent to participate

Not applicable.

## Authors' contributions

All authors contributed to study concept and design; data acquisition; data analysis and interpretation; drafting of the manuscript; critical revision of the manuscript intellectual content and statistical analysis.

## Figures and Tables

**Table 1.** Patients characteristics.

(*n* = 104)	Characteristics	*N* (%)
	Age	
	Median age	62 (32–86)
	<70 years	81 (77.9%)
	≥70 years	23 (22.1%)
	Gender	
	Female	54 (51.9%)
	Male	50 (48.1%)
	ECOG performance status	
	0–1	66 (63.5%)
	2–3	37 (35.6%)
	Subsite	
	IHC	57 (54.8%)
	EHC	38 (36.5%)
	GB	9 (8.7%)
	Status	
	Locally advanced	18 (17.3%)
	Metastatic	86 (82.7%)
	Baseline albumin	
	<3.5	11 (10.6)
	≥3.5	30 (28.6%)
	Missing data	63 (60.6%)
	Median CA19-9	180
	Missing data	38 (36.5%)
	Bone metastasis	
	No	84 (80.8%)
	Yes	20 (19.2%)
	Biliary obstruction	
	No	75 (72.1%)
	Yes	26 (25%)
	Missing data	3 (2.9%)

**Table 2.** First-line regimens.

Chemotherapy regimens	*N* (%)
GC	83 (79.8%)
5-FU based	12 (11.5%)
Single-agent	9 (8.7%)

**Table 3.** Univariate and multivariate analysis for OS.

	Univariate analysis			Multivariate analysis	
Variable	HR (95% CI)	*p* value		HR (95% CI)	*p* value
Age					
<70 years	1				
>70 years	1.43 (0.82–2.51)	0.203			
Gender					
Female	1.00			1.00	
Male	1.88 (1.19–2.98)	0.007		4.18 (1.62–10.8)	0.003
Staging					
Locally advanced	1				
Metastatic	1.43 (0.74–2.74)	0.279			
Primary site					
IHC	1				
EHC	1.06 (0.64–1.75)	0.81			
GB	1.42 (0.63–3.20)	0.39			
ECOG performance status					
0–1	1.00				
2–3	1.58 (0.98–2.53)	0.058			
Bone metastasis					
No	1.00			1.00	
Yes	1.82 (0.99–3.34)	0.051		3.53 (1.18–10.5)	0.023
CA19-9					
<Median	1				
>Median	1.37 (0.84–2.24)	0.202			
Baseline albumin					
≥3.5	1.00				
<3.5	4.73 (1.96–11.4)	0.001			
Biliary obstruction					
No	1.00			1.00	
Yes	2.15 (1.24–3.71)	0.006		4.28 (1.75–10.44)	0.001
Chemotherapy					
GC	1.00			1.00	
5-FU based	2.64 (1.31–5.3)	0.006		6.24 (1.41–27.6)	0.016
Singe-agent	6.27 (2.36–16.6)	<0.001		7.22 (1.30–39.8)	0.023
